# Development of Multiscale Composite with Hybrid Natural Nanofibers

**DOI:** 10.3390/ma15134622

**Published:** 2022-06-30

**Authors:** Javed A. K. Tipu, Syed Usman Rafiq, Muhammad Arif, Tariq Feroze, Hafiz Waqar Ahmad, Umer Masood Chaudry, Tea-Sung Jun, Adnan Aslam Noon

**Affiliations:** 1 Department of Mechanical Engineering, International Islamic University, Islamabad 44000, Pakistan; javed.ahmed@iiu.edu.pk (J.A.K.T.); rafiq.usman@iiu.edu.pk (S.U.R.); m.arif@iiu.edu.pk (M.A.); 2 Military College of Engineering (MCE) NUST, Risalpur Campus, Risalpur 23200, Pakistan; tariq.feroze@nust.edu.pk; 3 School of Mechanical Engineering, Sungkyunkwan University, Suwon 16419, Korea; waqar543@skku.edu.pk; 4 Department of Mechanical Engineering, Incheon National University, Incheon 22012, Korea

**Keywords:** nanofibers, natural nanofibers, composite, composition, electrospun, solution

## Abstract

Natural nanofibers are widely used in the field of medicine, but the low strength of these nanofibers is one of the major concerns. A number of factors, importantly the composition, affect the strength of natural nanofibers. The purpose of the current study is to ascertain the effect of the composition of natural nanofibers on the strength of hybrid composites formed using these nanofibers. Hybrid composites formed using 32% volume glass fibre with optimized volume fraction of 0.5% of pure Cellulose Acetate (CA), and 0.5% CA + Hemp Seed (HS) for this study to carry out the analysis. Hybrid composites were produced with vacuum-assisted resin transfer molding (VARTM) by collecting natural nanofibers, produced using the electrospinning process, over glass fiber mats. The electrospinning process was carried out with 12 kV, 10 cm tip to the collector gap, and 12% concentration of the solution. The tensile strength of the hybrid composites was measured using the universal testing machine (UTM). The results showed that the diameter of the electrospun nanofiber varied between 50 and 1400 nm and was affected by solution concentration, voltage, tip-to-collector distance, flow rate, and inclusion of HS in CA. The inclusion of HS in CA, for all compositions, decreased the fiber diameter and caused the formation of beads prominently at higher concentrations. Hybrid composites formed from nanofibers produced using CA and HS showed higher elastic modulus (232 MPa) and tensile strength (20.4 GPa) as compared with nanofibers produced using CA only (elastic modulus = 110 MPa and 13.7 GPa).

## 1. Introduction

Nanofibers with different physical properties can be produced from a range of synthetic and natural polymers to suit different application requirements. Nanofibers produced from natural polymers/fibers have a range of biomedical applications, such as scaffolds for tissue engineering, cardiovascular implants, repair of articular cartilage, urethral catheters, mammary prostheses, vascular grafts, penile prostheses, artificial skin, and adhesion barriers. Natural fibers are generally derived from three sources: plants, minerals, and animals. They are characterized by low density, moderate tensile strength (200–1000 MPa), stiffness in the range of 20–60 GPa, and low cost [1,2]. Around 2000 classes of plants are used as a source for obtaining these fibers [3]. Plant fibers can be classified into nonwood and wood fiber. The recycling process of plant fibers is easier than that of mineral fibers [4]. Overall, plant fibers have superior stiffness and strength compared with animal fibers, with the exception of silk fibers. These characteristics make natural fibers the most suitable fibers for the manufacturing of bioproducts [5,6].

Plant fibers and plant fiber composites have drawn the attention of research in the last two decades [6]. The nanofibers used in the biomedical and healthcare sector are particularly made of biodegradable or biocompatible materials [7]. Whey protein nanofibers have been used in regenerative medicine applications. For the development of new therapeutic wound dressing, electrospun hydrophilic nanofiber mats are used [8]. Gelatin nanofiber mats from the *Centella asiatica* (L.) plant are used for its healing ability [9]. Nanofiber from chitosan/polyethene and oxide/green is used to decrease inflammation and increase the speed of recovery of wound healing [10]. Nanofibers from sorghum and zein nanofiber are used for practical uses in medical applications and controlling bacterial growth [11]. Nanofibers from basil seed mucilage are used for different applications, such as packaging film production and bioactive encapsulation [12]. Deep eutectic solvent-zein nanofibers developed in the range of 350 ± 50 nm through the electrospinning process show exceptional hydrophilic properties [13]. Yue Jioa et al. produced self-healing hydrogels using chemical and physical functionalization. The polymerization process consisted of 2-2-6-6-tetramethylpiperidin-1-yl)oxyl, polyacrylic acid. The research claimed high mechanical properties, viscoelasticity, and increased self-healing. Different techniques, such as self-assembly, template-based synthesis, polymerization, sonochemical synthesis, and electrospinning, are employed to produce natural micro-/nanofibers [14]. Electrospinning is, however, one of the more preferred techniques that is employed to easily produce natural nanofibers. Among various advantages, electrospinning allows controlled porosity of the electrospun material, making of 3D structures, ease in the functionalization of fabricated nanofibers, and ease of fabrication of very thin fibers with a bigger surface area.

**Figure 1:**
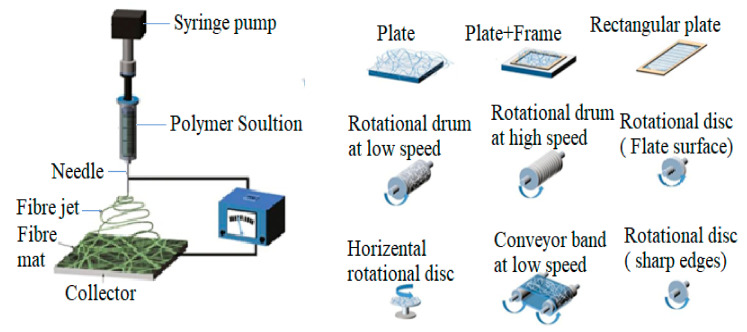
Principle electrospinning setup and collector types [15], while rotating collectors can reduce the randomness of the nanofibers.

A typical electrospinning process, shown in Figure 1, consists of a syringe pump set up to provide high voltage (1 to 30 kV) to induce charge on the drops of the polymer solution and a needle to release the polymer solution in the form of a fiber jet with a fastidious feeding rate to be collected on a collector. Due to Rayleigh’s uncertainty, the structure of a fiber can be pretentious. When the jet initially emits (a very short duration) from the tip, it follows a straight path, and then the looping, curling, winding, and bending of the jet happen [15], forming a nanofiber over the collector. Collectors can be different types, rotating or fixed, placed with different ranges 5–30 cm away from the core electrode. This distance is not fixed; it can be varied, depending on the spinning condition. Generally, stationary collectors are used to collect randomly oriented nanofibers. 

Electrospinning can be used to produce nanofibers from a number of natural polymers. Kebede, T.G. et al. fabricated good-quality blended nanofibers, with an average diameter of 232.87 ± 59.35 nm, from water-soluble proteins extracted from Moringa stenopetala seeds using electrospinning. The concentrations and parameters used include 10% (*w*/*v*) protein/polyvinyl alcohol (PVA) solution in 3% formic acid, a voltage of 15 kV, 12.5 cm tip-to-collector distance, and a flow rate of 5 μL/min [16]. F. Kurd et al. fabricated nanofibers with an average diameter of 179–390 nm using electrospinning from basil seed mucilage (BSM) [12]. N. Angel et al. produced nanofibers, with an average diameter of 404–1346 nm, using electrospinning from cellulose acetate using acetone solvent. The electrospinning process parameter included a needle (22 gauge), flow rate (2 mL/h), and voltage (9 to 15 kV) [17]. Sailing Zhu et al. manufactured an elastomer composite from carbon-nanotube-doped silylated cellulose nanocrystal. The resulting product showed high electrical conductivity along with high strength and was tested as a strain sensor [18]. S.O. Han et al. electrospun nanofibers from cellulose and studied the deacetylation of CA through different solvent systems and showed that by changing the composition of the mixed solvent, the average diameters of the CA nanofibers could be controlled from 160 to 1280 nm [19]. Silvestri et al., using the electrospinning method, produced nanofibers from graphene oxide (GO), gum arabic (GA), and polyvinyl alcohol (PVA) [20]. S.T. Sullivan et al. produced nanofibers from whey proteins. Aqueous whey protein solutions, whey protein isolate (WPI), and beta-lactoglobulin (BLG) were electrospun into nanofibers, with an average diameter of 312 to 690 nm, using a polymer, polyethylene oxide (PEO) [21]. Sofia El-Ghazali et al. successfully developed artificial blood vein using electrospinning nanofibers from the solution of poly (ethyleneglycol-co-1,4-cyclohexane di-methylene-co-isosorbide terephthalate) and poly (1,4 cyclohexane dimethylene-co-isosorbide terephthalate) [22].

M. Kowalczyk et al. showed that a composite formed using cellulose possesses has higher storage modulus as compared with composites formed using a PLA matrix [23]. R. Panneerdhass et al. fabricated epoxy polymer hybrid composites from luffa and found the range of mechanical properties to be: a compressive strength of 26.66 to 52.22 MPa, a tensile strength of 10.35 to 19.31 MPa, a flexural strength of 35.75 to 58.95 MPa, and an impact energy of 0.6 to 1.3 joules [24]. S. Ochi et al. developed biodegradable “green” composites from Manila hemp fiber bundles and a starch-based emulsion-type biodegradable resin. The tensile and flexural strengths of the composites increased with increasing fiber content up to 70%. The tensile and flexural strengths of the composites were found to be 365 and 223 MPa, respectively. Fabrication with emulsion-type biodegradable resin contributed to the reduction in voids in the composites [25,26]. S.H. Teng et al. electrospun uniform composite fibers, with a diameter of 60 nm, from collagen −30 wt. % HA composite solutions in an organic solvent [27,28].

The literature review shows that the use of natural nanofibers is generally limited to the medical engineering field because of the low strength. It is therefore important to see how the strength of natural nanofiber composites can be improved. The paper investigates the effect of adding hemp seed (HS) on the mechanical properties of a hybrid nanocomposite of “cellulose acetate (CA)”. A nanocomposite in the form of nanofibers was developed through the electrospinning process from CA (matrix) and a mixture of acetone and acetic acid (solvent). HS was initially used for reinforcement. Hybrid composites were later developed using a glass fiber and epoxy polymer matrix with different volume fractions to carry out the tensile testing. The electrospun nanofiber composites were collected over glass fiber mats, and a hybrid composite was manufactured using VARTM. After the hybrid composite fabrication, five rectangular plates having dimensions of 165 mm × 19.5 mm for tensile testing were machined by CNC milling to investigate the mechanical properties of the hybrid composites.

## 2. Materials and Methods

### 2.1. Fabrication of Nanofibers

**Figure 2:**
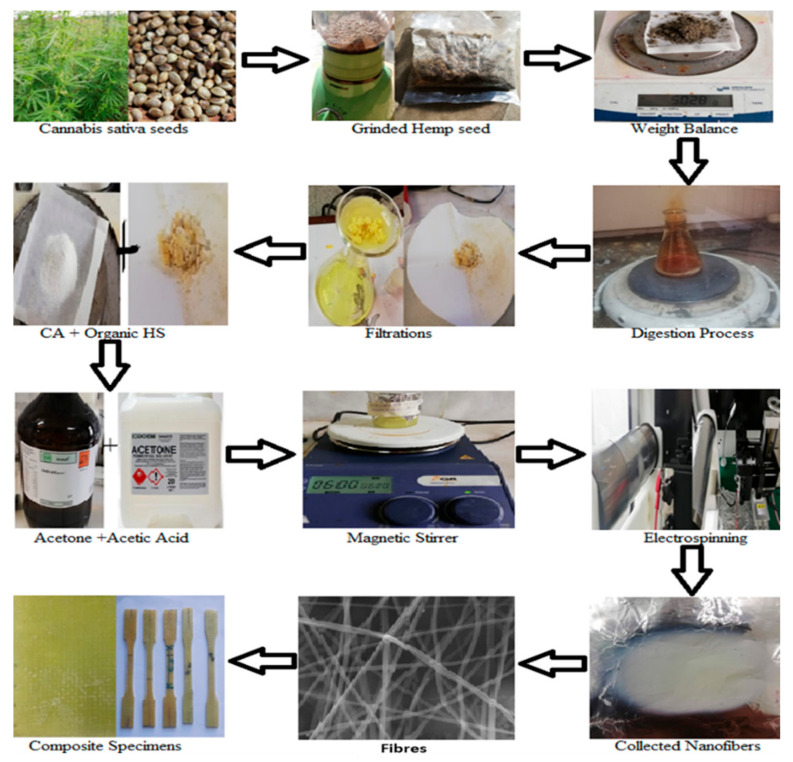
Overall Experimental process of nanofibers.

Hemp seed was cleaned of foreign matters (damaged material, soil, stones), dried in an oven at 80 °C, and ground into powder. The extraction of the organic compound from hemp seeds was carried out using the digestion process. CA with a density of 1.28 g/cm^3^ and HS with a density of 0.68 gm/cm^3^ were used to prepare the polymer solution with a 1:1 solution of acetone and acetic acid (as a solvent) at a 12% *w*/*v* concentration. A constant amount of solvent (acetic acid and acetone) was used with each sample for ease of comparison. The amount of HS and CA was varied as per details in Table 1. The mixed polymer solution of all samples was stirred at 500 rpm at room temperature for 24 h using magnetic stirrers. The overall procedure is depicted in Figure 2.

The homogeneous polymer solution was loaded into a syringe and placed in the syringe pump to electrospin horizontally on the collector. Electrospinning processes were carried out at room temperature, and the details of the major parameter and equipment are given in Table 2. When the experiment was performed once, the end of the needle was cleaned regularly to keep the formation of big drops at the end of it.

#### 2.1.1. Initial Screening: Selection of Parameters

Early analysis showed that the nanofibers could not be produced using a voltage above 16 kV and a flow rate below 1 mL/h. Best results were found at a voltage of 10 kV and a flow rate of 2 mL/h. Three polymer solution concentrations (6%, 8.3%, 12%) of CA and HS were used with different HA contents (0, 0.3, 0.5, 0.7, 0.8, 1) *w*/*v* %. The best nanofibers were found to be formed with a 12% solution concentration and an HS content of 0.5 *w*/*v* %. Further analysis was carried out with these settings, as given in Table 2.

#### 2.1.2. Characterization and Sample Collection

**Figure 3:**
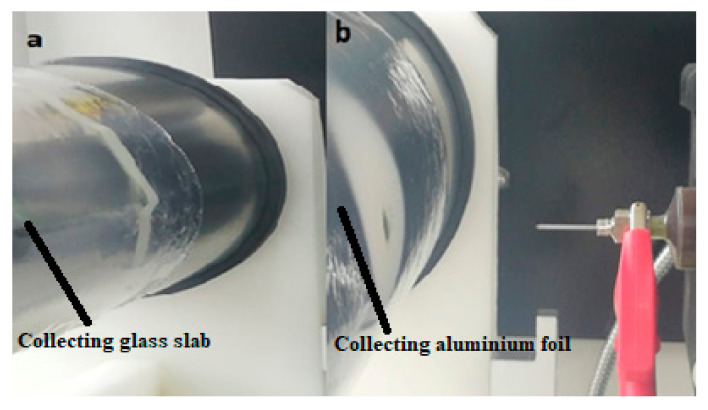
(**a**) Glass holder to collect samples for microscopes (**b**) for SEM Analysis.

Nanofiber samples were collected using different methods. Two dissimilar collectors were used: collector a: a slide was used to achieve the optical microscopy sample, and collector b: aluminum foil with a larger area than that of collector a was used to collect the nanofibers. For the microscopy sample, a microscope slide was placed on the collator drum, and collector b was used to collect nanofibers for SEM analysis, as shown in Figure 3.

#### 2.1.3. Optical Microscopy

In the optical microscopy study, it was observed that the polymer solution of cellulose acetate and hemp seed (CA + HS) fabricated the nanofibers. CA + HS nanofibers were electrospun on the microscopy slide for 15 to 30 min depending on the composition.

#### 2.1.4. Morphology Study

The morphology study of nanofibers/nanocomposites was performed using a scanning electron microscope (SEM), JEOL JSM-7500F (JEOL Ltd., Tokyo, Japan) at an acceleration voltage between 0.5 and 30 kV depending on the sample. The sample was prepared for SEM analysis, and each sample was sputter-coated using a magnetron sputtering apparatus. For the magnetron sputtering, small pieces of the sample were carefully cut and coated with gold (up to 5 nm) using a CY-VTC-16-SM (MTI Corporation, Richmond, CA, USA) magnetron sputtering coated apparatus to prepare the SEM samples.

### 2.2. Fabrication of Nanohybrid Composites

A hardener (bisphenol-A epoxy) and AMG 21 resin were used as base matrices for the fabrication of nanohybrid composites. AMG 21 has excellent thermal and mechanical properties, as given in Table 3. The recommended standard mix ratio of this resin given in the manufacturer manual is 100:38 by volume and 100:33 by weight.

E-glass macrofiber mats (woven) were used as a reinforcing material, and the physical and mechanical properties are shown below in Table 4.

Another reinforcement (nanomats) was produced from cellulose acetate + hemp seed/acetic acid + acetone polymer solution.

#### 2.2.1. Volume Fraction Calculations

The numbers of layers were calculated for the glass fiber to obtain 32% glass fiber in the epoxy matrix. The dimension of the single glass fiber was 19 cm × 19 cm to meet the VARTM mold cavity requirement. A single layer was cut, and the weight was measured to be 14.5 ± 0.5 g. To calculate the number of layers, Equation (1) was used:

(1) $$\eta \;\mathrm{layers}=\frac{\rho \mathrm{gf}\times \mathrm{Vgf}}{\mathrm{ma}}$$

where gf = glass fiber density = 2.58 g/cm^3^, Vgf = volume fraction of the glass fiber mats (16%, 24%, 32%, 40%); and ma = mass per unit of glass fiber = 0.04017 g/cm^3^.

The layers of glass fiber were approximated to be 3, 5, 7, and 9 for 16%, 24%, 32%, and 40%, respectively. The calculated mass of CA + HS and volume of CA + HS/A + AA solution for producing the required nanomat volume fraction are given in Table 5.

#### 2.2.2. Vacuum-Assisted Resin Transfer Molding (VARTM)

Electrospun natural nanofibers were collected on the glass fiber surface through the electrospinning process. The composites were fabricated using a vacuum-assisted resin transfer molding (VARTM) method. The fiber composites were manufactured using bisphenol-A (Ampreg 21) epoxy resin with woven E-glass fiber volume fractions of 16%, 24%, 32%, and 40%. The optimized 32% glass fiber composites were further strengthened using the following CA + HS nanofiber mats as discussed before for producing nanostrengthened hybrid composites, 0.1%, 0.2%, 0.5%, and 1% volume fractions of randomly distributed CA + HS nanofiber mats.

Three pieces of mold were used in the manufacturing of the composite: a female square steel ring and upper and lower male plates. Male plates were provided with O-ring grooves to provide a stable vacuum during the process. First, the lower male plate was positioned on a mold stand. After this, the female square steel ring was placed over the lower male plate, and then before closing the mold with an upper male plate, eight fiberglass mats equivalent to the required fiber volume fraction were placed inside. The mold assembly was fastened using C-clamps to prevent vacuum breaks. A suction and a riser port of the mold were connected through pipes to the resin reservoir and vacuum pump, respectively. After setting up the VARTM assembly, the resin–hardener was mixed with a volume ratio of 100:38 and placed at the suction port. Then the vacuum pressure valve was opened slowly, and a pressure of 80 psi was maintained until the resin filled the mold. Both the suction and riser ports were closed after filling, and then the resin was cured for 24 h at room temperature.

A similar procedure was followed for producing nanomat-strengthened hybrid composites except that the alternative layers of glass fiber mats and electrospun nanofiber mats were placed. Dissimilar volume fractions (0.1%, 0.2%, 0.5%, and 1%) of randomly oriented CA + HS polymer nanofiber mats were used with 32% glass fiber for manufacturing the CA + HS nanomats, which strengthened (random nanofiber) hybrid composites. A volume fraction of 0.5% of CA + HS polymer was selected to produce multiscale nanohybrid composites. Their results showed that the addition of HS by more than 2% with the CA + HS/acetone + acetic acid solution drastically increased the viscosity of the CA + HS/acetone + acetic solution, resulting in beaded electrospun fibers with larger diameters.

### 2.3. Mechanical Characterization

**Figure 4:**
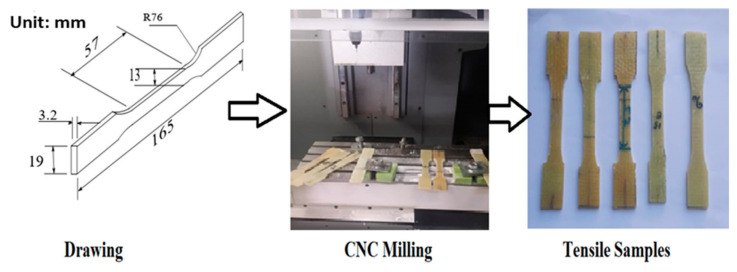
Testing procedure.

A sample for the tensile test was prepared from a preform manufactured using VARTM. The test was conducted according to the American Society for Testing and Materials (ASTM) standard. Five testing specimens (165 mm × 19 mm) with a thickness of 3.2 mm (Figure 4) were machined (CNC milling) and tested from a single VARTM composite preform respective to ASTM D638:2010, ASTM D790:2010, and ASTM D256:2010. The tensile test was carried out with a universal tensile testing machine. The recorded result was then used to obtain the tensile properties. A 10 kN load cell was used for testing. The tests were performed at a speed of 2 mm/min. All the specimens failed within the gauge length, which provided the validity of the tensile tests.

## 3. Results and Discussion

In this research study, randomly continuous nanofibers in the form of nanocomposites from CA and HS were obtained by the mixed solvent of acetone/acetic acid using an electrospinning process. The polymer solution was analyzed by birefringence and electrospun. The collected fibers were easily observed by the naked eye and characterized by SEM. The mechanical properties of the nanohybrid composites, produced from CA and HS mats with E-glass fiber using the resin–hardener by VARTM, were investigated. The conclusion and results obtained are explained below.

**Figure 5:**
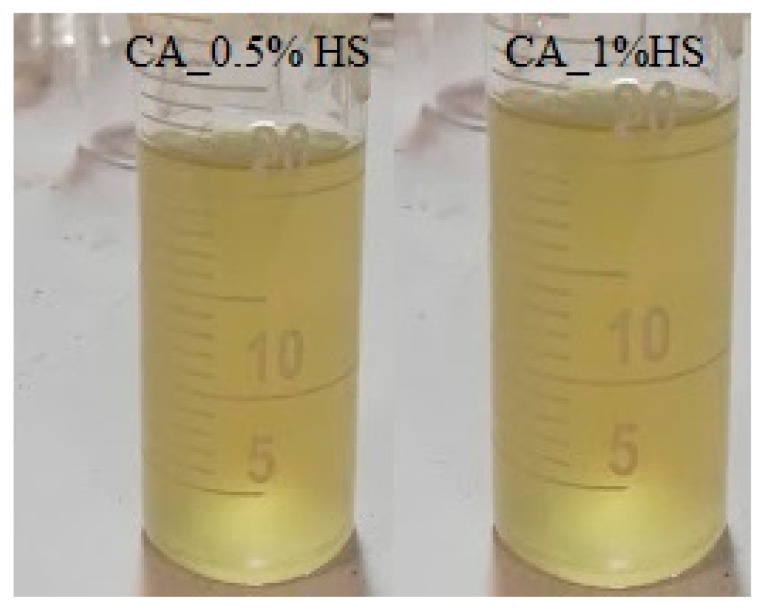
Birefringence flow test.

After preparing the 12% polymer solution concentration with different (0, 0.3, 0.5, 0.7, 0.8, and 1 *w*/*v* %) HS contents, the flow birefringence test was conducted, which verified the dissolution of HS in the solution (Figure 5).

### 3.1. Electrospinning Process Parameters and Effects

**Figure 6:**
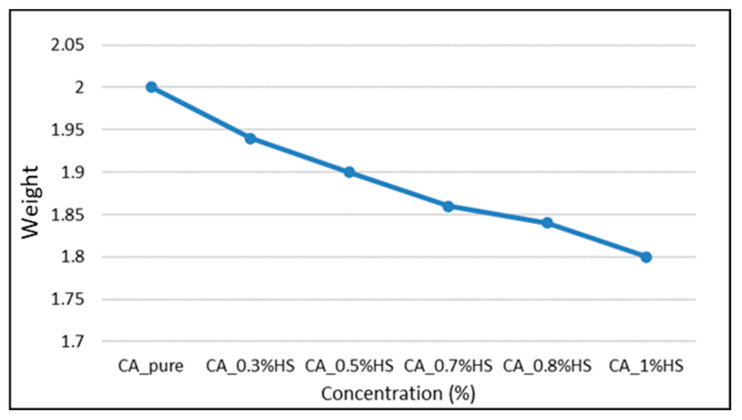
Tensile testing samples’ average weight.

It is already established that the electrospinning process can be affected by several parameters, such as HS content, collection time, tip-to-collector distance, and flow rate. Therefore, the tensile test samples of glass fiber mats showed different weights (Figure 6).

**Figure 7:**
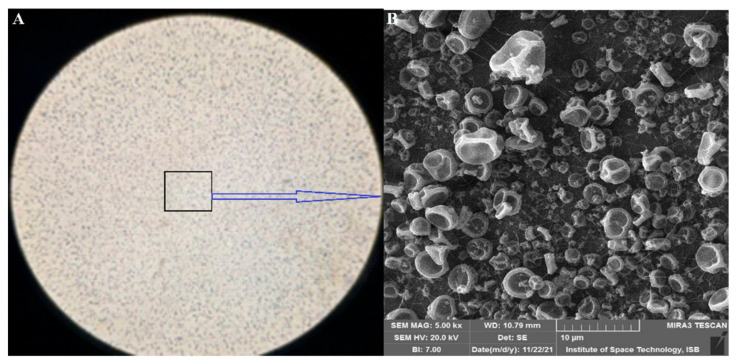
Picture of electrospun CA + HS 10% higher than 16 kV and a flow rate of 1 mL/h (**A**) electrospun substrate; (**B**) SEM image of electrospun splashing beads.

The phenomenon of increased density with increased concentration was also observed by C. Mit-Uppatham et al. [29]. Nozzle-to-collector distance affected the fabrication and orientation of the fibers. The production of nanofibers at 6 to 10 cm was more viable than at larger distances (14 cm). Lower voltages (15 kV or less) gave smooth production of nanofibers. Voltages higher than 16 kV produced spraying action of polymers (Figure 7).

### 3.2. Electrospun Pure CA Nanofibers

**Figure 8:**
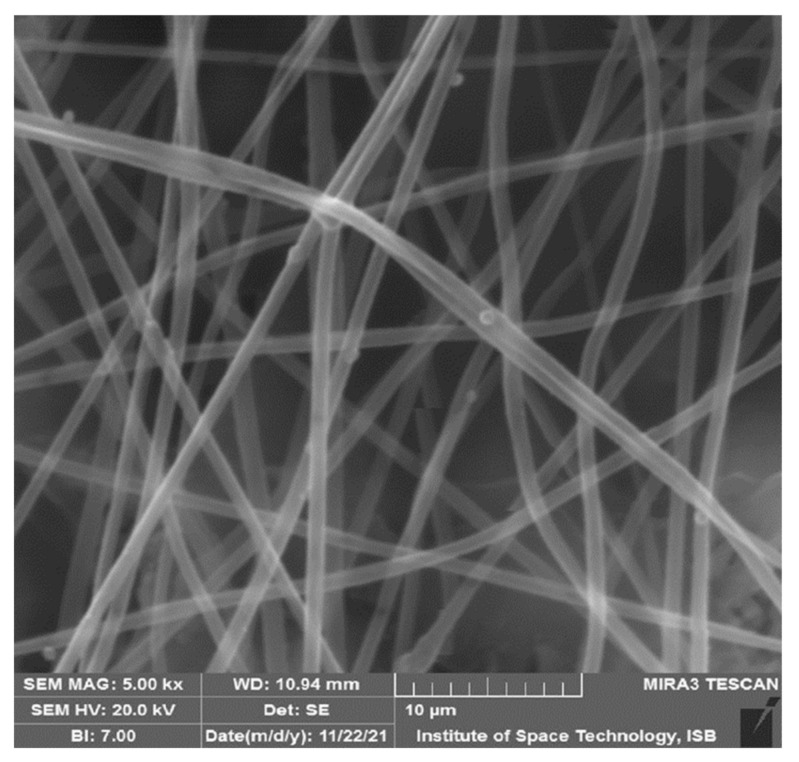
Pure CA SEM image.

Pure CA nanofibers were fabricated using an electrospinning process with a 12% concentration of the solution. The electrospinning process’s parameters were a voltage of 12 kV, a nozzle-to-collector distance of 10 cm, a flow rate of 2 mL/h, and a collecting time of 25 to 30 min. Randomly oriented nanofibers were collected on a rotating drum, and parameters remained the same for the rest of the experiments. Nanofibers produced with optimized parameters were examined. The diameter of pure CA solution was in the range of 800 ± 30 um (Figure 8).

### 3.3. Electrospun CA + HS Nanofibers

**Figure 9:**
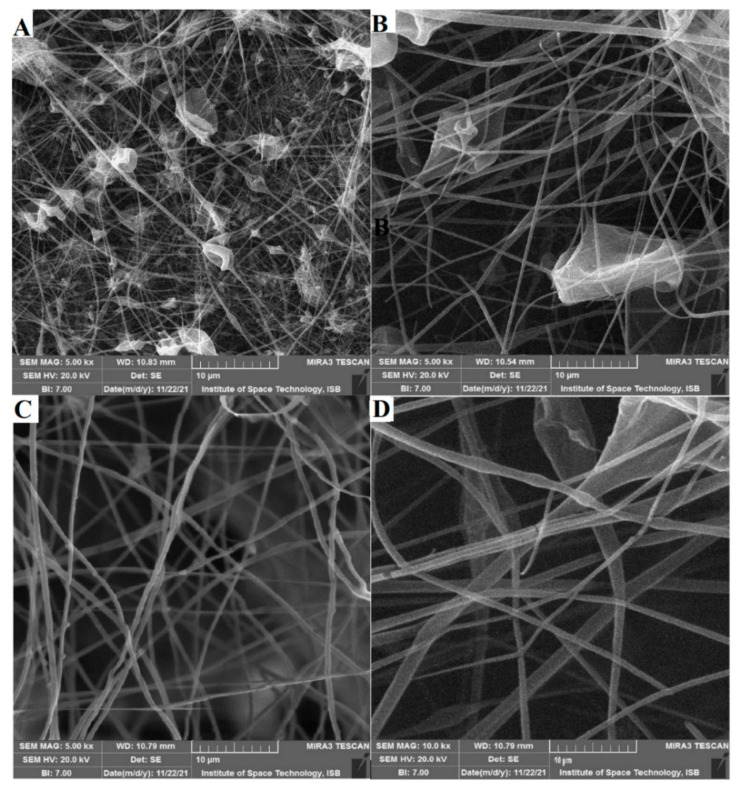
SEM images of nanofibers (voltage: 12 kV; gap: 6 cm): (**A**) CA_0.3% HS, (**B**) CA_0.5% HS, (**C**) CA_0.7% HS, and (**D**) CA_0.8% HS.

The CA with HS nanofibers in the form of the nanocomposite was obtained using the same electrospinning parameters (voltage of 12 kV, flow rate of 2 mL/h, and tip-to-collector distance of 10 cm). The HS content in the polymer solution below 0.5% and above 1% resulted in the fabrication of beaded nanofibers. The HS contents of 0.7% and 0.8% produced nanocomposite fibers with lesser beads are shown in Figure 9.

### 3.4. Effect of Diameter on Nanofiber Size

**Figure 10:**
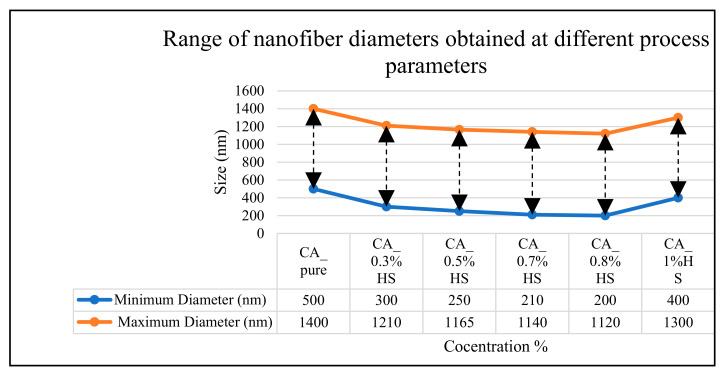
Range of nanofiber diameters produced from pure CA and CA + HS at different process parameters.

SEM analysis showed that the diameter of the nanocomposite fibers formed using CA increased by increasing the concentration of CA and ranged between 900 and 1400 nm. The effect of concentration on the diameter of the nanofibers obtained using CA and HS was not as pronounced as that produced with CA and varied between 50 to 500 nm for the range of concentrations used in this study. The smallest-diameter (50 nm) bead-free nanofibers were found with 0.8% concentration. Several measurements were made for each composition to measure the minimum and maximum diameters to analyze the effect of the concentration of CA and HS contents, as shown in Figure 10. It can be seen that the diameter of the fiber decreased with the increase in HS concentrations. The phenomenon of reduced diameter with increased polymer concentration is as also claimed by Julia [30].

### 3.5. Mechanical Properties

The tensile specimen was tested using 10 KN load cells. The results for each category of the composites are given in the following paragraphs.

#### 3.5.1. Glass Fiber Composites

**Figure 11:**
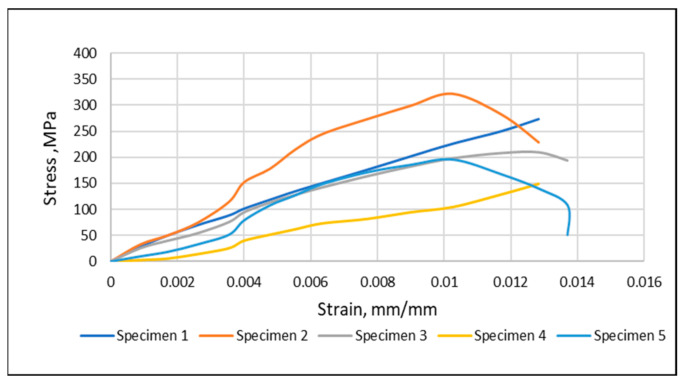
Tensile test result of five test specimens—32% volume fraction glass fiber.

The tensile strength (Figure 11) of the glass fiber mats slowly increased for fraction volumes up to 32%. Then the glass fiber mats improved the load resistance of the composites by transferring the load from the resin to the glass fiber. It may be related to the limited penetration of the resin to the glass fiber mats. Therefore, the 32% volume fraction of the glass fiber composite was selected for further study.

**Figure 12:**
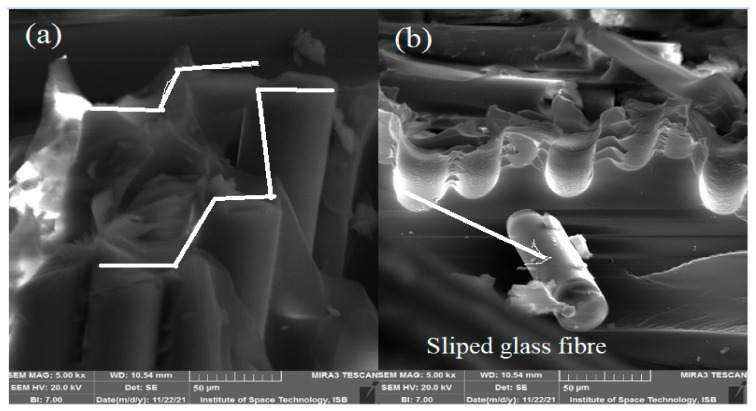
SEM image of 32% glass fiber. (**a**) Stepwise breaking of glass fiber (**b**) Glass fiber slippage.

A morphological study (SEM) of a tested (32%) glass fiber composite showed the broken surface of the glass. The crack initiation in the matrix-rich region travelled to the glass fibers, and then the glass fiber separated along the interface region. Low bondage at the interface region of the glass fiber and matrix due to length size mismatch provided low resistance to load, and failure occurred. Figure 12a shows the stepwise breaking of glass fibers, which infer that, initially, one fiber was broken, and then load proceeded to the others. Figure 12b shows a glass fiber slippage due to the low resistance provided by the interface region between the glass fiber and the matrix. A SEM study suggested a weak matrix and glass fiber interface due to length size mismatch between molecular chains of the matrix and glass fiber.

#### 3.5.2. Random CA Nanofiber-Strengthened Hybrid Composite

**Figure 13:**
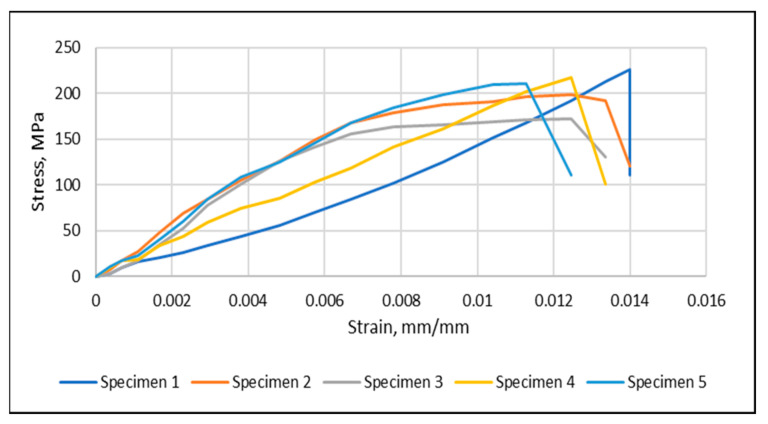
Tensile test result—0.5% volume fraction pure CA.

An elastic modulus and tensile strength of a randomly oriented (CA) nanofiber-mat-strengthened hybrid composite (volume fraction 0.5%) are shown in Figure 13. The tensile strength increased with the addition of randomly oriented (CA) nanofiber mats. CA increased the elastic modulus and tensile strength of the 32% glass fiber composite by 18.25% and 85.11%, respectively.

**Figure 14:**
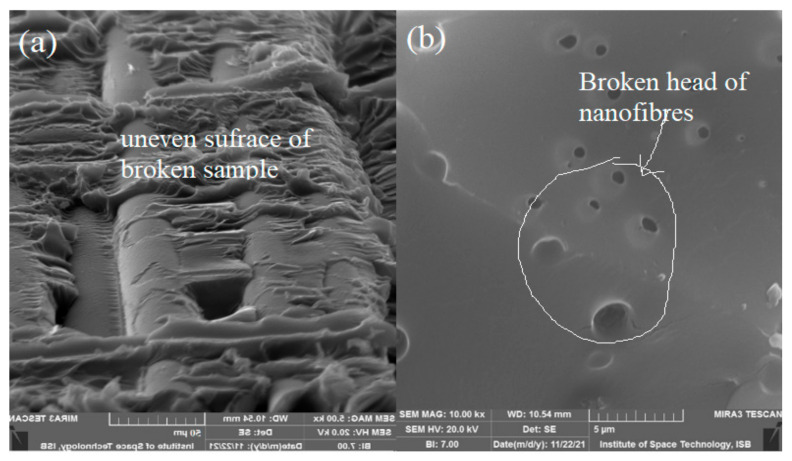
SEM image of tensile test of 0.5% CA + 32% glass fiber composite. (**a**) Plane breakage of a composite (**b**) broken fiber holes.

A SEM image of a glass fiber composite reinforced with 0.5% CA randomly oriented nanofibers showed a plane breakage of a composite rather than the slippage of glass fibers (Figure 14a). The increased strength inferred that the CA nanofibers provided more resistance to applied force at the interface region. Figure 14b shows broken fiber holes during the tensile test, which reduced the large mismatch among the glass fiber matrix and molecule chains and improved the strength of the interface region.

#### 3.5.3. Random CA + HS Nanofiber-Strengthened Hybrid Composite

**Figure 15:**
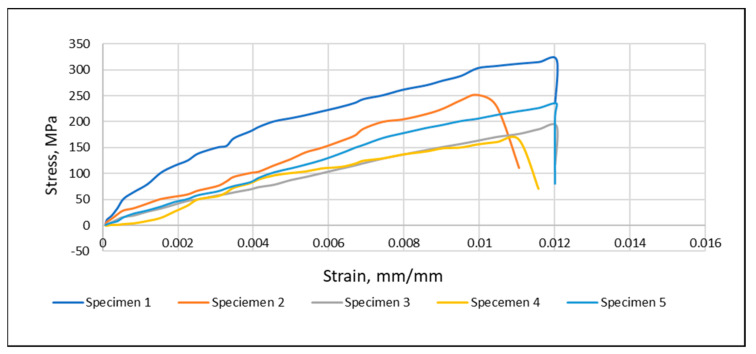
Tensile test result—0.5% volume fraction (CA + HS).

Neat glass fiber composites were strengthened using 0.5% CA + HS nanofiber mats. The nanofibers were collected over a glass fiber substrate, and a nanohybrid composite was developed using VARTM. For the production of CA + HS nanofiber mats, HS 0.7% *w*/*v* was used. The tensile result of random (CA) nanofiber mats and a CA + HS random nanofiber is shown in Figure 15. The random CA + HS nanofiber increased the elastic modulus and tensile strength of the 32% glass fiber composite by 48.9% and 109.8%, respectively. This increase is due to the reduction of the CA + HS nanofiber diameter. Table 6 shows the average tensile strength properties of nanofiber mat hybrid composites of 32% glass fiber, pure cellulose acetate, and cellulose acetate with hemp seed organic compound.

**Figure 16:**
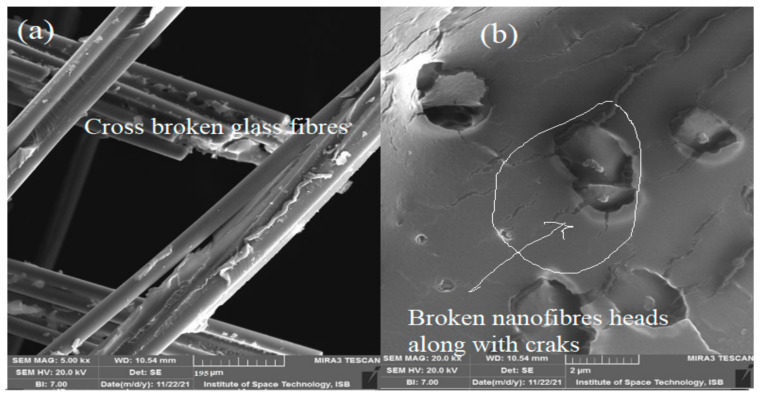
SEM image of tensile test of 0.5% CA + HS + 32% glass fiber composite. (**a**) Breaking of glass fibers in the cross direction (**b**) Pullout of 0.5% (CA + HS) nanofibers.

It was observed that mats produced with 0.5% (CA + HS) nanofibers improved the tensile strength of fibers better than fiber mats produced with 0.5% CA. The reason may be the smaller size of CA + HS nanofibers than that of CA nanofibers. The same amount of volume provided more networks of nanofibers into the composites. Another aspect is the smaller aspect ratio of the nanofibers, which results in more strength as compared with CA fibers of larger aspect ratio. Breaking of glass fibers in the cross direction without shattering into them pieces was observed in 0.5% (CA + HS) nanofibers (Figure 16a). Moreover, the pullout of 0.5% (CA + HS) nanofibers occurred along with the cracks, as shown in Figure 16b.

## 4. Conclusions

Electrospun natural nanocomposites were successfully developed from CA as well as CA and HS and analyzed for size and morphology using scanning electron microscopy in this research study. VARTM was used for the fabrication of hybrid composites, and the mechanical properties (Young’s modulus, strain, and tensile strength) were obtained using the universal testing machine (UTM). The results showed that suitable nanofibers were produced when the electrospinning process was carried out with a 12 kV voltage, 10 cm collector gap, and 12% concentration of polymer solution. The range of the diameter of the fibers reduced considerably from 900–1400 to 50–500 nm with the addition of HS as compared with the use of CA only. Furthermore, the formation of beaded fibers was noticed when the concentration of HS was more than 2% and less than 0.5%.

## Figures and Tables

**Table 1:** Each sample composition with 12% concentration.

Samples		Electrospinning Solution 12% *w*/*v*			
		Solute (CA + HS)			Solvent (g) [1:1] *v*/*v*
No.	HS (% *w*/*v*)	Solute (g)	HS (g)	CA (g)	
**1**	0	2.00	0.000	2.000	16.66
**2**	0.3	2.00	0.060	1.940	16.66
**3**	0.5	2.00	0.100	1.900	16.66
**4**	0.7	2.00	0.140	1.860	16.66
**5**	0.8	2.00	0.160	1.840	16.66
**6**	1	2.00	0.200	1.800	16.66

**Table 2:** Electrospinning parameters/equipment details.

Parameter/Equipment	Detail
**Syringe volume**	1–50 mL
**Stainless steel needle (G22)**	Connected to positive electrode
**Syringe pump (HO-NFES-043C)**	Control flow rate at 0.001–90 mL/min
**Voltage source**	0–50 kV
**Speed collector drum (D = 100 mm, L = 350 mm)**	(0–140 rpm) Connected to negative electrode
**Voltage**	5–15 kV
**Flow rate**	0.5–3 mL/h
**Nozzle-to-collector distance**	6–14 cm

**Table 3:** Physical and thermal properties of Ampreg 21 (resin and hardener).

Standard Hardener/Resin					
**Initial Mixed Viscosity (cP)**	Gel Time (min)	Cured Density (g/cm^3^)	Tensile Strength (MPa)	Tensile Modulus (GPa)	Demold Time (min)
1194	21	1.148	72.7–81.3	3.3–4.3	143

**Table 4:** E-glass (woven) properties.

Property	Density (g/cm^3^)	Young’s Modulus (GPa)	Tensile Strength (MPa)	Tensile Elongation (%)
Value	2.6	81.3	1.72	2.4

**Table 5:** Volume fraction for different concentrations of CA + HS/acetone + acetic acid.

Volume Fraction of Nanofiber	Mass of CA + HS (Gram)	Volume of CA + HS/A + AA Solution 12%
0.1	0.226	3.67
0.2	0.398	5.47
0.5	0.914	10.85
1	1.774	19.82

**Table 6:** Average tensile strengthen properties of nanofiber mat hybrid composites.

Composite	Tensile Strength (MPa)	% Increase in Tensile Strength	Young’s Modulus (GPa)	% Increase in Young’s Modulus
**32% glass fiber**	110.8		13.7	
**0.5% Pure CA**	205.10	85.11%	16.2	18.25%
**0.5% CA + HS**	232.5	109.8%	20.4	48.9%

## Data Availability

Data are contained within the article.

## References

[1] Jain R., Shetty S., Yadav K.S. (2020). Unfolding the electrospinning potential of biopolymers for preparation of nanofibers. J. Drug Deliv. Sci. Technol..

[2] Osanloo M., Arish J., Sereshti H. (2019). Developed methods for the preparation of electrospun nanofibers containing plant-derived oil or essential oil: A systematic review. Polym. Bull..

[3] Guna V., Ilangovan M., Adithya K., Akshay Koudshik C.V., Srinivas C.V., Yogesh S., Nagananda G.S., Venkatesh K., Reddy N. (2019). Biofibers and biocomposites from sabai grass: A unique renewable resource. Carbohydr. Polym..

[4] (2000). Book reviews. Phytomorphol. Int. J. Plant Morphol..

[5] Pickering K.L., Aruan Efendy M.G., Le T.M. (2016). A review of recent developments in natural fibre composites and their mechanical performance. Compos. Part A Appl. Sci. Manuf..

[6] Gowda B. (2007). Economic Botany.

[7] Stepanyan R., Subbotin A., Cuperus L., Boonen P., Dorschu M., Oosterlinck F., Bulters M. (2016). Nanofiber diameter in electrospinning of polymer solutions: Model and experiment. Polymer.

[8] El-Ghazali S., Kobayashi H., Khatri M., Phan D.-N., Khatri Z., Mahar S.K., Kobayashi S., Kim I.-S. (2021). Preparation of a Cage-Type Polyglycolic Acid/Collagen Nanofiber Blend with Improved Surface Wettability and Handling Properties for Potential Biomedical Applications. Polymers.

[9] Sikareepaisan P., Suksamrarn A., Supaphol P. (2007). Electrospun gelatin fiber mats containing a herbal—*Centella asiatica*—extract and release characteristic of asiaticoside. Nanotechnology.

[10] Sadri M., Arab-Sorkhi S., Vatani H., Bagheri-Pebdeni A. (2015). New wound dressing polymeric nanofiber containing green tea extract prepared by electrospinning method. Fibers Polym..

[11] Yang S.B., Yoo S.H., Rabbani M.M., Kim I.K., Oh W., Han S.I., Yeum J.H. (2017). Incorporation of Sorghum Extract into Electrospun Zein Nanofibers and Their Characterization. J. Nanosci. Nanotechnol..

[12] Kurd F., Fathi M., Shekarchizadeh H. (2017). Basil seed mucilage as a new source for electrospinning: Production and physicochemical characterization. Int. J. Biol. Macromol..

[13] Khatri M., Khatri Z., El-Ghazali S., Hussain N., Qureshi U.A., Kobayashi S., Ahmed F., Kim I.S. (2020). Zein nanofibers via deep eutectic solvent electrospinning: Tunable morphology with super hydrophilic properties. Sci. Rep..

[14] Kenry, Lim C.T. (2017). Nanofiber technology: Current status and emerging developments. Prog. Polym. Sci..

[15] Huang Z.-M., Zhang Y.-Z., Kotaki M., Ramakrishna S. (2003). A review on polymer nanofibers by electrospinning and their applications in nanocomposites. Compos. Sci. Technol..

[16] Kebede T.G., Dube S., Nindi M.M. (2018). Fabrication and characterization of electrospun nanofibers from *Moringa stenopetala* seed protein. Mater. Res. Express.

[17] Angel N., Guo L., Yan F., Wang H., Kong L. (2019). Effect of processing parameters on the electrospinning of cellulose acetate studied by response surface methodology. J. Agric. Food Res..

[18] Zhu S., Sun H., Lu Y., Wang S., Yue Y., Xu X., Mei C., Xiao H., Fu Q., Han J. (2021). Inherently Conductive Poly(dimethylsiloxane) Elastomers Synergistically Mediated by Nanocellulose/Carbon Nanotube Nanohybrids toward Highly Sensitive, Stretchable, and Durable Strain Sensors. ACS Appl. Mater. Interfaces.

[19] Han S.O., Youk J.H., Min K.D., Kang Y.O., Park W.H. (2008). Electrospinning of cellulose acetate nanofibers using a mixed solvent of acetic acid/water: Effects of solvent composition on the fiber diameter. Mater. Lett..

[20] Silvestri D., Mikšíček J., Wacławek S., Torres-Mendieta R., Padil V.V.T., Černík M. (2019). Production of electrospun nanofibers based on graphene oxide/gum Arabic. Int. J. Biol. Macromol..

[21] Sullivan S.T., Tang C., Kennedy A., Talwar S., Khan S.A. (2014). Electrospinning and heat treatment of whey protein nanofibers. Food Hydrocoll..

[22] El-Ghazali S., Khatri M., Hussain N., Khatri Z., Yamamoto T., Kim S.H., Kobayashi S., Kim I.S. (2021). Characterization and biocompatibility evaluation of artificial blood vessels prepared from pristine poly (Ethylene-glycol-co-1,4-cyclohexane dimethylene-co-isosorbide terephthalate), poly (1, 4 cyclohexane di-methylene-co-isosorbide terephthalate) nanofibers and their blended composition. Mater. Today Commun..

[23] Kowalczyk M., Piorkowska E., Kulpinski P., Pracella M. (2011). Mechanical and thermal properties of PLA composites with cellulose nanofibers and standard size fibers. Compos. Part A Appl. Sci. Manuf..

[24] Panneerdhass R., Gnanavelbabu A., Rajkumar K. (2014). Mechanical Properties of Luffa Fiber and Ground nut Reinforced Epoxy Polymer Hybrid Composites. Procedia Eng..

[25] Ochi S. (2006). Development of high strength biodegradable composites using Manila hemp fiber and starch-based biodegradable resin. Compos. Part A Appl. Sci. Manuf..

[26] Prahasti G., Edikresnha D., Rezeki Y.A., Munir M.M., Khairurrijal K. (2019). The Synthesis and Characterization of Composite Electrospun Fibers of Polyvinylpyrrolidone and Shell Extract of Melinjo (*Gnetum gnemon* L.). Mater. Today Proc..

[27] Teng S.-H., Lee E.-J., Wang P., Kim H.-E. (2008). Collagen/hydroxyapatite composite nanofibers by electrospinning. Mater. Lett..

[28] Dong Y., Ghataura A., Takagi H., Haroosh H.J., Nakagaito A.N., Lau K.-T. (2014). Polylactic acid (PLA) biocomposites reinforced with coir fibres: Evaluation of mechanical performance and multifunctional properties. Compos. Part A Appl. Sci. Manuf..

[29] Mit-Uppatham C., Nithitanakul M., Supaphol P. (2004). Ultrafine Electrospun Polyamide-6 Fibers: Effect of Solution Conditions on Morphology and Average Fiber Diameter. Macromol. Chem. Phys..

[30] Hernández-Vargas J., González-Campos J.B., Lara-Romero J., Ponce-Ortega J.M. (2014). A Mathematical Programming Approach for the Optimal Synthesis of Nanofibers through an Electrospinning Process. ACS Sustain. Chem. Eng..

